# The Aging Vaginal Microenvironment: A Communication Toolkit

**DOI:** 10.3390/amh71020012

**Published:** 2026-05-10

**Authors:** Laneshia Conner, Lirisha Tuladhar

**Affiliations:** College of Social Work, University of Kentucky, 631 Patterson Office Tower, Lexington, KY 40506, USA

**Keywords:** aging vagina, clinical toolkit, microbial shifts, health professionals, older women

## Abstract

**Background:**

The vagina undergoes important changes across the life course that are shaped not only by hormonal transitions but also by shifts in the vaginal microbial environment. Despite growing interest in the vaginal microbiome, research has disproportionately centered reproductive-aged populations, leaving the aging vagina comparatively understudied.

**Objective:**

This article examines the aging vagina through a life-course lens, with emphasis on microbial and clinical transitions associated with midlife and older adulthood.

**Key Content:**

The article highlights menopause-related changes and approaches for reducing stigma and missed clinical opportunities. Particular attention is given to menopause-related declines in estrogen, reduced glycogen availability, increased vaginal pH, and accompanying changes in microbial balance, as well as their relationship to dryness, irritation, genitourinary symptoms, and susceptibility to adverse outcomes. The article also provides health professionals with a practical educational framework for symptom recognition, patient communication, vaginal health assessment, menopause-related education, stigma reduction, and prevention of missed clinical opportunities.

**Conclusions:**

Positioning the aging vagina within life-course and microbial-health frameworks can strengthen prevention, improve symptom recognition, and support more ageinclusive, informed, and responsive care for older women.

## 1. Introduction

Sexual well-being remains a meaningful dimension of health across the life course. However, routine sexual assessment and sexual health education are often absent in care for older adults [1–4]. In practice, sexuality is frequently dismissed as irrelevant to later life or addressed only in the context of dysfunction, despite ongoing needs for prevention, counseling, and accurate interpretation of bodily changes [5–7]. This disconnect creates a practical problem: older women may experience pain, bleeding, discomfort, or heightened vulnerability to infection without receiving clear, non-stigmatizing guidance [7,8].

Meanwhile, scientific and clinical knowledge on later-life sexual health has expanded considerably—particularly regarding how menopause reshapes the vaginal microenvironment through hormonal and tissue changes that interact with local microbial ecology [9–12]. These alterations can affect comfort, susceptibility to injury, and disease vulnerability. Yet such knowledge is rarely integrated into community-based sexual health education or the conversational tools practitioners use when discussing issues with older women [13,14].

As a result, current practice is often variable and idiosyncratic. Whether older women are asked about sexual concerns, receive anticipatory guidance, or are referred appropriately may depend on a practitioner’s comfort, training background, and time constraints rather than on a consistent, evidence-aligned approach [15–17]. A structured framework can reduce this variability by offering a shared standard for what should usually happen. At the same time, it can allow flexible, case-based tailoring when client values, history, culture, or clinical presentation warrant a different approach. In this sense, structure supports—rather than replaces—professional judgment.

Social workers functioning as public health educators are well positioned to narrow this gap. Viewed as approachable professionals, they build close client relationships, guiding older adults through medication side effects, alerting them to potential impacts on sexual health, and offering therapeutic alternatives to help adapt to bodily and functional changes [5,18]. They bring strengths in holistic assessment, relational communication, culturally responsive practice, and stigma reduction—skills that directly address barriers suppressing later-life sexual health dialogue [19]. Yet without a clear, stepwise approach that translates menopause-related microenvironment knowledge into usable assessment and education steps, practice remains inconsistent and dependent on individual discretion.

Among women who are aging and at different stages of menopause or are post-menopausal, there are compositional shifts to the vagina that have clinical implications for health professionals when providing education and care. For those professionals who are less driven by the biomedical model of care, it is essential to identify and understand the shifts in the vaginal microbiome. The vaginal microbiome changes across the life course, reflecting the interaction of biological aging with social, behavioral, and structural factors. In reproductive-age individuals, vaginal microbial communities are often characterized by *Lactobacillus* dominance, particularly species such as *L. crispatus, L. iners, L. gasseri,* and *L. jensenii* [20]. While these shifts are frequently described in biomedical terms, they also have broader implications for how aging individuals experience vaginal health, sexual intimacy, bodily change, symptom disclosure, and access to appropriate care [21].

These shifts are important because they can affect how aging individuals experience vaginal and urinary health in everyday life. *Lactobacillus* species help support a balanced vaginal environment. When these bacteria decline and other bacteria become more common, individuals may be more likely to experience dryness, irritation, discomfort during sex, urinary symptoms, and infections. These symptoms can influence quality of life, sexual wellbeing, help-seeking behavior, and perceptions of aging. *Lactobacillus* species help maintain a protective vaginal environment, while lower levels of *Lactobacillus* and greater microbial diversity are linked to vaginal dryness, irritation, inflammation, painful sex, urinary discomfort, recurrent urinary symptoms, and increased risk of vaginal or urinary infections [21–24]. Thus, changes in the vaginal microbiome may contribute to the physical symptoms and quality-of-life concerns often reported during and after menopause.

Howard and Jenson [19] and others [20–24] provide a useful foundation to distinguish between elements that are evidence-based, such as menopause-related biological change, vaginal microbiome shifts, symptom burden, sexual wellbeing, and healthcare access barriers, and the proposed innovation of integrating these elements of biological aging with social dimensions. This integrated social science model of the aging vagina specifically aligns biological aging with lived experience.

To address this gap, this paper proposes a microenvironment-informed practice framework for social work health educators and other human service professions. This framework supports consistent, evidence-aligned conversations about the aging vagina without adding extra workload, providing up-to-date direction while preserving professional autonomy [19]. It emphasizes normalizing later-life sexuality, integrating menopause-related physiological and microbial shifts into education, and using concrete tools to improve communication and personalize support. Informed by the sexual health model [25] and drawing on applications of single-case research, these tools include a reproductive history questionnaire, an arts-based self-figure drawing activity, and aging-tailored labeled genital imagery. The sections that follow first summarize menopause-related changes in the vaginal microenvironment and explain why microorganisms matter for education and prevention. They then delineate gaps in current practice, before presenting the framework alongside implementation considerations, anticipated benefits, and potential criticisms.

## 2. Materials and Methods

### 2.1. Menopause, the Vaginal Microenvironment, and Why Microorganisms Matter

Menopause alters the vaginal microenvironment through hormonal and tissue shifts that interact with local microbial ecology [7,26]. Social work health educators require a foundational understanding of these changes to deliver clearer education and more effective prevention messaging.

### 2.2. Menopause-Related Tissue Changes and Sexual Comfort

Postmenopausal hormonal changes reduce vaginal elasticity and lubrication, rendering tissues more fragile and lowering the pain threshold during consensual sexual activity. Consequently, postmenopausal women are more susceptible to genital injuries overall [6]. This has critical implications for education, as many interpret such pain or bleeding as personal failure or “dysfunction.” A life-course perspective helps reframe these experiences as normal aging processes that affect resilience and healing capacity [27]. An apt analogy likens postmenopausal vaginal changes to those in aging skin or joints: reduced hormonal support decreases resilience, heightening susceptibility to irritation or injury without implying pathology. This framing fosters non-stigmatizing conversations while creating space to discuss care strategies and prevention.

### 2.3. The Microenvironment Lens: Linking Physiology and Microorganisms

The microenvironment lens underscores that risk and comfort are shaped locally, connecting age-related physiological changes to microbial shifts and challenging assumptions of uniform sexual resilience across the life course [28,29]. Microorganisms thus become practically vital for education and assessment; variations in the vaginal microbiome extend far beyond biological trivia. As previously discussed, menopausal changes in older women can alter vaginal ecology in ways that influence disease risk, prevention, diagnosis, and treatment within personalized sexual and reproductive health approaches.

Notably, to enhance feasibility and acceptability during framework development, we incorporated preliminary qualitative input from health professionals and older women through IRB-approved formative qualitative work. This study was approved for exemption (#102568) at the authors’ institution. The exemption was granted because the study involved anonymous survey data, minimal-risk interviews, and no private identifiable information was collected or reported. Health professionals completed semi-structured interviews on barriers to later-life sexual health conversations and desired educational tools. Older women participated in semi-structured reproductive health history discussions and arts-based self-figure drawing activities, followed by group discussions. We used these inputs to revise the stepwise protocol, simplify explanations of microenvironment changes, and strengthen trauma-informed, permission-based language.

### 2.4. The Development of Her Kit

An important part of concept development for a product is market discovery, the process of identifying and analyzing current products. Seven companies were identified that offer health models (e.g., skills trainers, condom kits, sexual health education tools, home care trainers, pelvic models), including models of the entire reproductive system as well as external genitalia. Common features of models were removable organs and/or the ability to disassemble parts. For example, a female pelvis model might have a removable uterus with fallopian tubes or the ability to disassemble the pelvis for a birthing demonstration. Some offer multiple flesh tone (usually two, light and dark). Most companies are authorized distributors and do not offer custom orders. Only one company stated that they offer customization. None of the models offer the ability to alter the existing models to aging or chronic condition specifications as suggested. Table 1 illustrates the model types and their ability to offer aging adaptations.

Upon review, and noting the lack of such a tool, the first author submitted an internal research proposal to support the development of the tool. Upon successful funding, the first author collaborated with the College of Fine Arts to develop prototypes of aging vagina mockups (examples) based on a medical literature review. A graphic illustrator designed the mockups based on a series of consultations with the research team, which included the first author and two professors in the art department. After several iterations of drafts, the final sketches were moved from Adobe Illustrator 2026 (version 30.0) to overlays used within Microsoft PowerPoint, facilitating the act of ‘building’ a custom vagina with various shapes and sizes of all the related tissue.

**Figure 1.**
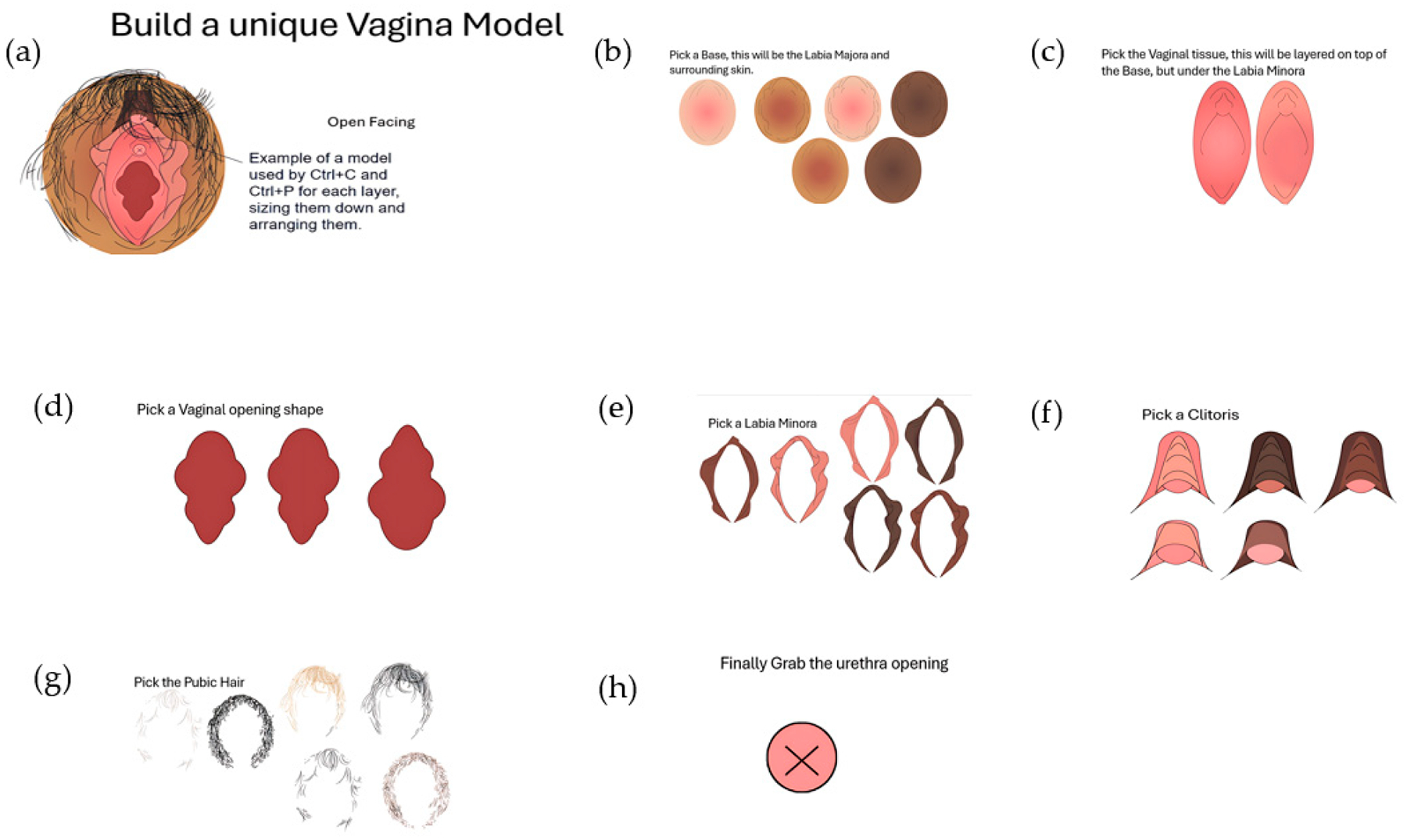
Guide for building a unique vagina model.

As seen in Figure 1, the illustrations were placed in a slide show. Each element could be changed in relation to participant’s feedback.

## 3. Discussion

This section presents a structured yet flexible framework to guide sexual health conversations with older women. Tailoring is essential when culture, trauma history, disability, or access barriers influence communication [30]. The goal is consistency without rigidity, as the framework supports a shared baseline for typical assessment and education while preserving clinical judgment and personalization.

### 3.1. A Microenvironment-Informed Practice Framework for Social Work Health Educators

Core Principles

1. Normalization and stigma reduction. Begin from the assumption that sexuality remains relevant in later life. Use nonjudgmental language. Treat questions about pleasure, pain, and risk as routine health topics.
2. Microenvironment-informed education. Explain that menopause can reshape local conditions (hormones, tissue, microbial ecology). Use this to contextualize symptoms and vulnerability without implying pathology.
3. Client-centered tailoring. Align education with the client’s values, relationship context, sexual practices, comfort level, and cultural background.
4. Trauma-informed and autonomy-respecting practice. Offer choices, ask permission, and avoid assumptions.
5. Prevention and appropriate referral. Pair education with clear “when to seek care” guidance, especially when symptoms suggest infection or other clinical issues.

**Figure 2.**
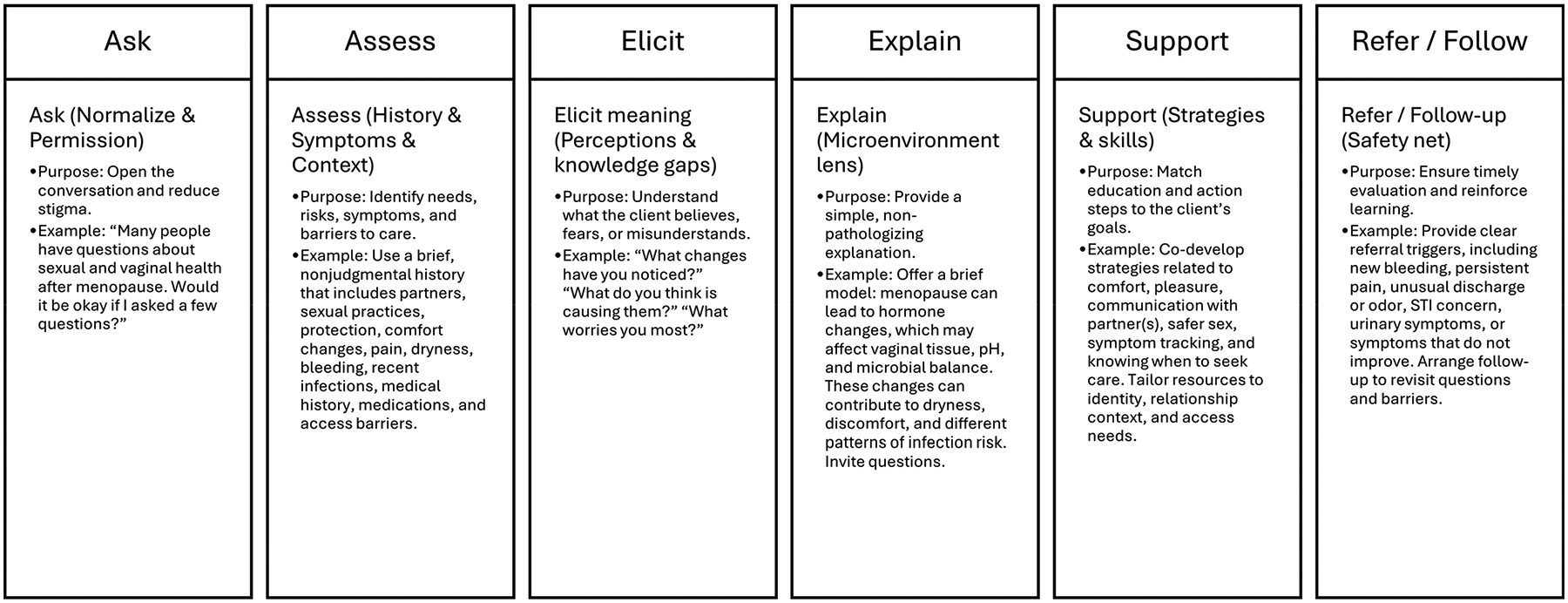
Sequence for Ask, Assess, Explain, Support, and Refer/Follow-up.

The framework follows a short, repeatable sequence: Ask, Assess, Explain, Support, and Refer/Follow-up (see Figure 2). It opens with a low-pressure normalization statement and permission-based question. Assessment then uses a reproductive and sexual history tool, alongside symptom prompts that address menopause-related comfort changes and risk factors. Next, the educator introduces a simple microenvironment lens, linking menopause-related tissue changes and microbial ecology to common experiences and prevention needs. The conversation then shifts to support, offering practical strategies—such as communication tips, comfort options, safer sex guidance, and resources—tailored to the client’s identity, partner status, and goals. Finally, it concludes with referral and follow-up: clear thresholds for clinical evaluation, brief follow-up questions, reinforced key messages, and strategies to address care barriers.

To put the framework into action, practitioners need concrete tools for assessment and education. A reproductive history questionnaire offers structured prompts to reduce missed topics and ease provider discomfort through clear language and logical flow. An arts-based genital self-figure drawing activity provides a client-friendly way to elicit perceptions, concerns, and knowledge gaps, especially for those lacking medical terminology [31]. Aging-tailored labeled genital imagery further strengthens education by depicting older bodies and enabling accurate, stigma-reducing explanations of anatomy and age-related changes.

For reference, the authors adapted nine questions from the publicly accessible Form 31- Reproductive History Questionnaire of the Women’s Health Initiative Clinical Trial and Observational Study SHARE [32]. They also collaborated with another unit to create illustrations that support, based on a medical literature review, the development of this imagery.

## 4. Implementation Considerations

This framework is designed for brief, real-world encounters. It can be used in primary care, community health programs, or social service settings. It can also be delivered over more than one session. In a short visit, educators can prioritize Step 1, Step 2, and a simplified Step 4. In longer encounters, the full sequence can be used, including the drawing activity.

Basic training is needed for consistent use. Educators should practice permission-based openings and nonjudgmental language. They should also learn a simple way to explain menopause-related microenvironment changes. Training should include traumainformed safeguards, because sexuality and bodies can be sensitive topics.

The tools should be offered as options, not requirements. The questionnaire can be self-completed or guided. The drawing activity should be voluntary. The imagery should be aging-tailored and culturally responsive. Documentation should be brief and respectful, and privacy should be protected. Clear referral triggers should be stated at the end of the conversation and revisited at follow-up.

## 5. Conclusions

Menopause alters the vaginal microenvironment through hormonal shifts, reduced tissue integrity, and microbial changes. This also leaves professionals with implications for comfort, vulnerability, and prevention. Yet this knowledge rarely informs routine sexual health assessment and education for older women. This paper fills that gap with a microenvironment-informed framework that ensures consistency without rigidity. Its stepwise protocol sets a baseline for conversations while allowing professional autonomy and client-centered adaptation.

Future studies should test feasibility across settings, acceptability among diverse older women, and outcomes like comfort discussing sexual health, knowledge gains, helpseeking, and referrals. With careful implementation and refinement, this guidance can normalize later-life sexual health and bolster prevention and well-being.

### Implications for Practice

Practice guidelines can strengthen evidence-based work and improve outcomes without adding extra burden [33]. When well designed, they provide up-to-date direction while preserving professional autonomy. In this paper, the proposed framework applies that same logic to later-life sexual health education by offering a clear sequence for routine care, while also leaving room for professional judgment and client-led tailoring.

A key implication is that microenvironment-informed education can become practical in everyday settings when paired with usable tools. The reproductive history questionnaire supports consistent assessment and reduces missed topics. The arts-based genital self-figure drawing activity helps clients express perceptions and concerns without requiring medical vocabulary. Aging-tailored labeled genital imagery supports accurate, stigma-reducing explanations and normalizes older bodies. Together, these tools operationalize the framework’s steps (Ask, Assess, Explain, Support, Refer/Follow-up) and reduce the likelihood that later-life sexual health is skipped because of discomfort, time pressure, or uncertainty about how to proceed.

These tools also extend to communication quality and equity. Research in health communication shows that tailoring improves message relevance when it reflects the audience’s norms, preferences, and lived context—including both “surface” features and “deep” features [30]. In this framework, aging-tailored imagery functions as a surfacestructure support, while permission-based language, stigma reduction, and attention to personal goals function as deep-structure supports. The result is a practice approach that can be consistent and still culturally responsive.

This protocol does not intend to standardize client experiences; rather, it standardizes the opportunity for care. Professional judgment remains central, and steps can be reordered or omitted as appropriate. Microorganism and microenvironment content is presented in plain language to inform prevention and interpretation, not to pathologize sexuality in later life. Finally, the arts-based activity is optional: it should be offered with consent and replaced with other elicitation strategies when not a good fit.

## Figures and Tables

**Table 1.** Market Discovery of Reproductive Health Training Models.

	Model Specifications			Offer Aging Specifications/Adaptability
Model Types (Company)	Pelvic Models	Vagina Models	Penis Models	Atrophy, Dermatitis, Hair (Presence/Absence), Color Changes, Size Changes, Chronic Conditio
Anatomical Pelvic Models (Mentone)	X	X		NO
Condom Training (Anatomy Warehouse)	X	X	X	NO
OB/GYN Education (Global technologies)	X	X	X	NO
Condom Trainers (3B scientific)	X	X	X	NO
Sex Education Products (Health EDCO)	X			NO
Interchangeable Catheterization and Enema Task trainer; In-Service Home Care Training Simulator (Laerdal)	X			NO
Genitalia (CAE)		X	X	NO

*Note*. The letter X is used to indicate the presence of specifications for the model.

## Data Availability

No new data were created.
